# Ancestry, admixture and fitness in Colombian genomes

**DOI:** 10.1038/srep12376

**Published:** 2015-07-21

**Authors:** Lavanya Rishishwar, Andrew B. Conley, Charles H. Wigington, Lu Wang, Augusto Valderrama-Aguirre, I. King Jordan

**Affiliations:** 1 School of Biology, Georgia Institute of Technology, Atlanta, GA 30332, USA; 2 PanAmerican Bioinformatics Institute, Cali, Valle del Cauca, Colombia; 3 BIOS Centro de Bioinformática y Biología Computacional, Manizales, Caldas, Colombia; 4 Biomedical Research Institute, Universidad Libre, Cali, Valle del Cauca, Colombia; 5 Regenerar - Center of Excellence for Regenerative and Personalized Medicine, Cali, Valle del Cauca, Colombia

## Abstract

The human dimension of the Columbian Exchange entailed substantial genetic admixture between ancestral source populations from Africa, the Americas and Europe, which had evolved separately for many thousands of years. We sought to address the implications of the creation of admixed American genomes, containing novel allelic combinations, for human health and fitness via analysis of an admixed Colombian population from Medellin. Colombian genomes from Medellin show a wide range of three-way admixture contributions from ancestral source populations. The primary ancestry component for the population is European (average = 74.6%, range = 45.0%–96.7%), followed by Native American (average = 18.1%, range = 2.1%–33.3%) and African (average = 7.3%, range = 0.2%–38.6%). Locus-specific patterns of ancestry were evaluated to search for genomic regions that are enriched across the population for particular ancestry contributions. Adaptive and innate immune system related genes and pathways are particularly over-represented among ancestry-enriched segments, including genes (*HLA-B* and *MAPK10*) that are involved in defense against endemic pathogens such as malaria. Genes that encode functions related to skin pigmentation (*SCL4A5*) and cutaneous glands (*EDAR*) are also found in regions with anomalous ancestry patterns. These results suggest the possibility that ancestry-specific loci were differentially retained in the modern admixed Colombian population based on their utility in the New World environment.

The arrival of Columbus in the New World precipitated a massive and sudden exchange of life forms between the American and Afro-Eurasian hemispheres. European colonization and trade in the Americas entailed a bidirectional exchange of numerous species of plants, animals and microbes, many of which until that time had been evolving separately for millions of years. The conquest and settlement of the Americas, along with the African slave trade, also included exchanges among a variety of human populations. These biological and cultural exchanges, which were often intentional although sometimes not so, had a tremendous impact on the trajectory of human history in the centuries that followed. Among other effects, the exchange of crops and livestock allowed for a major increase in the global population, whereas the introduction of microbes that cause infectious disease had devastating effects on the naïve populations to which they were introduced.

The historian Alfred Crosby referred to this massive transfer of life as the *Columbian Exchange* in his seminal book of the same name^1^. Numerous dimensions of the Columbian Exchange have been studied since the concept was first introduced, and these ideas have received renewed attention with the recent publication of the book *1493: Uncovering the New World Columbus Created* by Charles Mann^2^. In particular, the ecological, economic and cultural-historical aspects of this era have been explored at length. One especially intriguing aspect of the Columbian Exchange that has not been addressed within this conceptual framework is the exchange that occurred at the level of human genome sequences.

The modern human species emerged out of Africa and spread throughout the world starting between 60–100,000 years ago^3^. Human evolution during this time was primarily characterized by migration and geographical isolation, followed by population divergence. Over tens-of-thousands of years, these processes gave rise to the major continental groups of human populations recognized today: African, European, Asian, Melanesian and American^4^,^5^. The trajectory of human evolution was turned upside-down during the course of the Columbian Exchange. Within the last 500 years, populations that were separated for many thousands of years were brought back together, and as tends to be the case whenever humans are placed in close proximity, they then began to exchange genes. This process of genetic admixture in the Americas has occurred over an extremely short time period in human evolution, for <1% of the time since modern humans first emerged from the African continent.

Admixture rapidly brings together population-specific (or enriched) alleles that have not previously co-existed in the same genetic background, and thereby can be considered to result in the creation of completely novel human genomes. Population-specific (enriched) alleles are sequence variants that evolved to population-characteristic frequencies *in situ* within ancestral populations’ endemic geographic regions. Some of these alleles may have drifted to high frequency within the ancestral populations by chance, whereas others are likely to have been swept to fixation based on selection pressures that were distinct to the ancestral populations’ environments^6^,^7^,^8^,^9^. Genes that mediate humans’ interaction with their environment, such as those that encode skin pigmentation and immune system related proteins, seem to have been particularly prone to adaptive evolution in ancestral regions. For example, there are several skin pigmentation genes with European-specific and/or Asian-specific alleles that are associated with lighter skin color^6^,^7^,^8^,^10^,^11^,^12^,^13^,^14^,^15^,^16^. A number of genes involved in the defense against infectious pathogens also evolved regional-specific alleles that are concordant with the ranges of particular pathogens^6^,^7^,^8^,^9^,^10^. At least three distinct genes have evolved population-specific alleles related to the defense against malaria in regions endemic for the disease^7^,^8^,^10^. In general, populations from pathogen-rich global regions, including regions of West Africa, East Asia and the Americas, encode a more diverse repertoire of immune receptors, and this is thought to be due to selection pressure to confront a wider variety of microbial pathogens^17^.

In this study, we sought to address what it means when genomic variants that have been separated for tens-of-thousands of years are suddenly brought back together over the course of a few hundred years. In other words, is the process of modern admixture somehow related to human health and fitness? Our working hypothesis is based on the well-supported axiom that specific allelic variants (SNPs) have evolved separately in ancestral human populations based on their regional-specific utility, *i.e.* their relationship to health and fitness in a particular environment. We posit that these pre-evolved ancestral population-specific alleles may have been selected in the modern admixed population based on their utility in the new environment. The new environment could be a new physical environment, for populations that were transported to new regions, and/or a new ecological environment based on the mixing of previously isolated human populations and their associated microbial fauna.

We evaluate these ideas here via a study of the relationship between ancestry, admixture and fitness in Colombian genomes. The Colombian population is a particularly interesting subject for study in this regard owing to its high levels of ethnic admixture^18^. The people of Colombia are richly diverse with substantial admixture between African, Native American and European ancestral populations (Table 1)^19^,^20^,^21^,^22^,^23^. In fact, it has been reported that Colombians show among the greatest extent of three-way continental admixture of all genetically characterized Latino/Hispanic populations^24^,^25^. We analyzed the ancestry and admixture patterns for whole genome sequences of 60 unrelated Colombians from Medellin, which were recently sequenced as part of the 1000 Genomes Project^26^,^27^. We then developed and applied a method to search for genomic regions that show anomalous patterns of admixture based on differential retainment of chromosomal segments from specific ancestral populations in the modern admixed population. These anomalous regions were interrogated for previously identified signatures of natural selection as well as for the functional and health-associated roles of the genes encoded therein. This approach yielded results indicating that there is substantial enrichment for ancestry-specific loci genome-wide, and these regions encode numerous genes involved in immune related functions along with genes previously implicated in selection for skin pigmentation and glandular development.

## Results

### Ancestry and admixture in Colombian genomes

**Figure 1:**
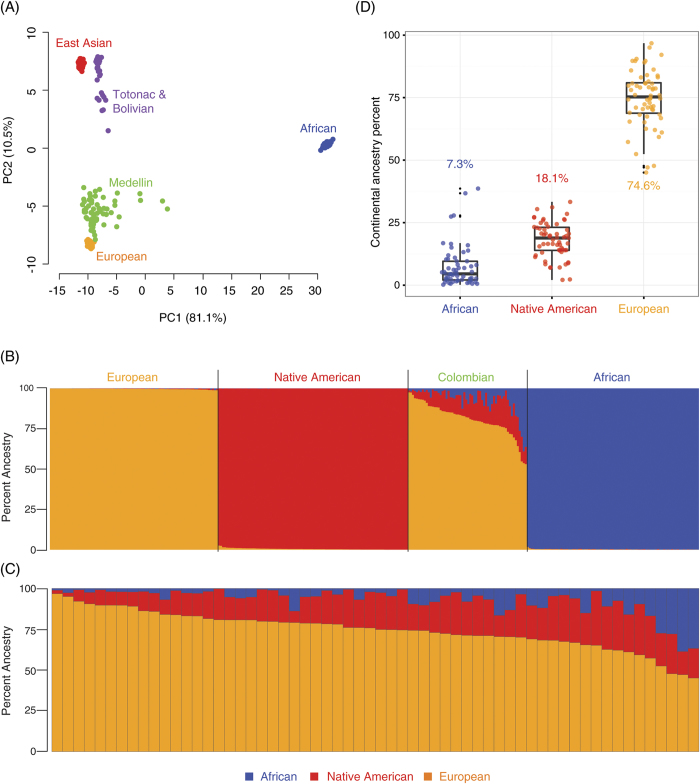
Ancestry and admixture patterns for Colombian genomes. (**A**) PCA of pairwise allele sharing distances among admixed Colombian genomes from Medellin compared with putative ancestral populations from Africa, the Americas, Asia and Europe. (**B**) Admixture plots showing ancestry proportions for three putative ancestral populations and the admixed Colombian genomes. (**C**) The lower panel shows only the Colombian genomes. (**D**) Ancestral admixture proportion distributions and averages for the Colombian genomes.

Admixed Colombian genome sequences from Medellin were compared with a number of world-wide populations that are likely to be closely related to Colombian ancestral source populations (Supplementary Fig. S1). To do this, pairwise allele (SNP) sharing distances were computed between all genomes and principal component analysis (PCA) was used to project the resulting pairwise distances (Fig. 1A and Supplementary Fig. S2). The first principal component (81.1% of the variation) shows clear separation between the African ancestral population and all other populations, whereas the Native American, East Asian and European ancestral populations are separated along the second principal component (10.5% of the variation). The Colombian genomes from Medellin appear most closely related to the European ancestral population, but extend outward along both principal component axes consistent with African and Native American admixture. The East Asian population is most closely related to the Totonac Native American population from Mexico followed by the Bolivian population, which shows higher levels of apparent European admixture.

The close clustering of the East Asian with the Bolivian Native American populations (Fig. 1A) is consistent with the relatively recent origin of Native Americans from Asia^28^ and suggests that Asian genomes are a viable surrogate for inferring Native American ancestry contributions at the continental level. In other words, when admixed Colombian genomes are analyzed in three-way continental level comparisons to ancestral source populations, including African and European populations, East Asian genomes are very likely to recover Native American ancestral genomic segments. Whole East Asian (CHB) genome sequences were used here, along with whole African (YRI) and European (CEU) genome sequences, for admixture analysis of the Colombian genomes in an effort to provide additional resolution beyond what is available from relatively sparse genotype data. An admixture plot showing the three ancestral genome clusters, together with the admixed Colombian genome cluster, is shown in Fig. 1B along with a plot expanding the Colombian genomes (Fig. 1C). The Colombian genomes show substantial variability in admixture patterns with different levels of contribution from ancestral populations. The percent ancestry values for individual genomes range from 1.2% African, 2.1% Asian (Native American) and 96.7% European to 36.8% African, 18.2% Asian (Native American) and 45.0% European. The admixture contributions to the Colombian genomes from ancestral populations are well outside the error levels seen based on the extent of false-positive ancestry assignment levels in the ancestral clusters (Fig. 1B). The average ancestry values for the admixed Colombian genomes from Medellin are 7.3% African, 18.1% Asian (Native American) and 74.6% European (Fig. 1D).

### Sex-specific admixture

**Figure 2:**
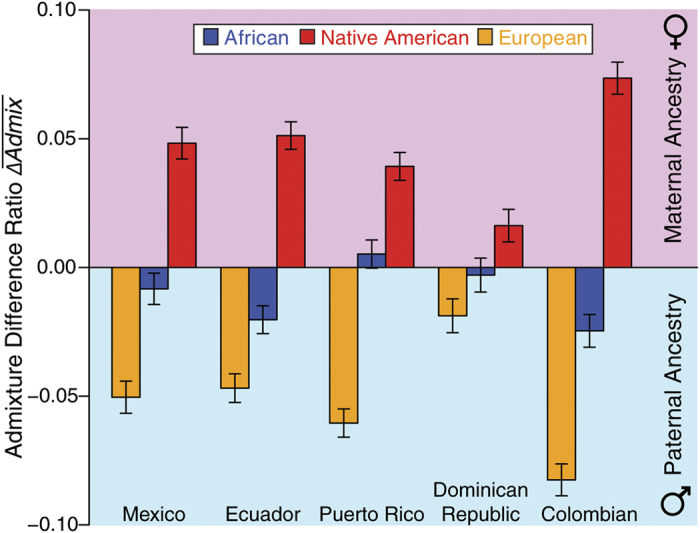
Sex-specific contributions to three-way genomic admixture in Latin American countries. For each ancestry component – African, Asian (Native American) and European – the normalized difference between the X chromosome ancestry fraction and the autosomal ancestry fraction is shown. Positive values in the plot indicate a relative excess of female-specific (maternal) ancestry for a given admixture component, whereas negative values indicate an excess of male-specific (paternal) ancestry.

A number of previous studies have uncovered sexual asymmetry in the contributions of different ancestral populations to admixed Latino genomes^20^,^21^,^22^,^23^,^25^. Admixed Latino genomes tend to show a relative excess of European paternal ancestry and proportionally greater Native American maternal ancestry. We evaluated the sexual asymmetry of admixture in the Colombian population by comparing ancestry contributions to the X chromosome versus the autosomes as described in the Materials and Methods. Since X chromosomes spend relatively more time along the female lineage, a relative excess of a specific ancestral admixture component in X chromosomes indicates a proportionally greater female (maternal) contribution for that ancestry. Conversely, a relative excess of a specific ancestral admixture component in autosomes indicates a proportionally greater male (paternal) contribution. Consistent with previous reports, the Colombian population shows highly sex-specific admixture patterns with predominantly European contributions to the male lineage and Native American ancestry along the female lineage (Fig. 2). Interestingly, this pattern is more pronounced for the Colombian population than for any of the other four Latino populations with which it was compared. The relative level of European male ancestry for the Colombian population is significantly greater than seen for that of the country with the next highest level (Puerto Rico; *P* = 1.8 × 10^−7^). Similarly, the relative level of Native American female ancestry was significantly higher for the Colombian population compared with the country with the next highest level (Ecuador; *P* = 1.2 × 10^−7^). The Colombian population also shows a smaller, but not insubstantial, excess of African ancestry along the male lineage (African paternal ancestry differs from 0 at *P* = 3.2 × 10^−14^).

### Population-wide admixture enrichment

**Figure 3:**
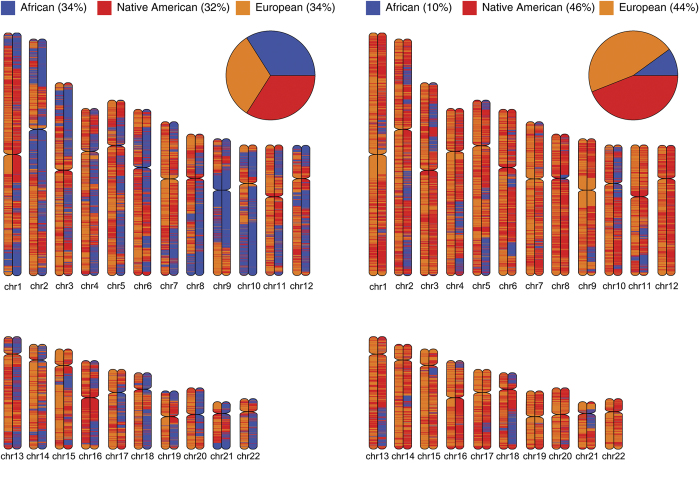
Regional (locus-specific) ancestry and admixture in Colombian genomes. Chromosome paintings showing the genomic distributions of loci with African, Asian (Native American) and European ancestry, along with their genome-wide ancestry proportions, are shown for two example Colombian individuals.

The three-way continental ancestry origins of individual chromosomal segments across the entire genome were determined for all 60 individuals in the Colombian population as described in the Materials and Methods. Chromosome paintings that show the locations of ancestry-specific segments across the chromosomes of two Colombian individuals are shown in Fig. 3; similar results for all 60 Colombian genomes analyzed here can be found in the Supplementary Video. As can be seen from these plots, along with Fig. 1B–D, individual Colombians vary substantially with respect to both their overall three-way continental ancestry contributions and their regional (locus-specific) ancestry origins. The locus-specific patterns of ancestry among the Colombian population were analyzed to search for anomalous genomic regions that are enriched for contributions from a particular ancestral population. To do this, the population-wide ancestry profiles of individual chromosomal segments were compared with the overall average ancestry contributions as described in the Materials and Methods. The boundaries of the individual chromosomal segments (loci) used for this ancestry enrichment analysis are defined by recombination maps as previously described^29^. There are 379,218 such loci genome-wide and the average length of an ancestry block is 7,542 bp (see loci size distribution in Supplementary Fig. S3).

**Figure 4:**
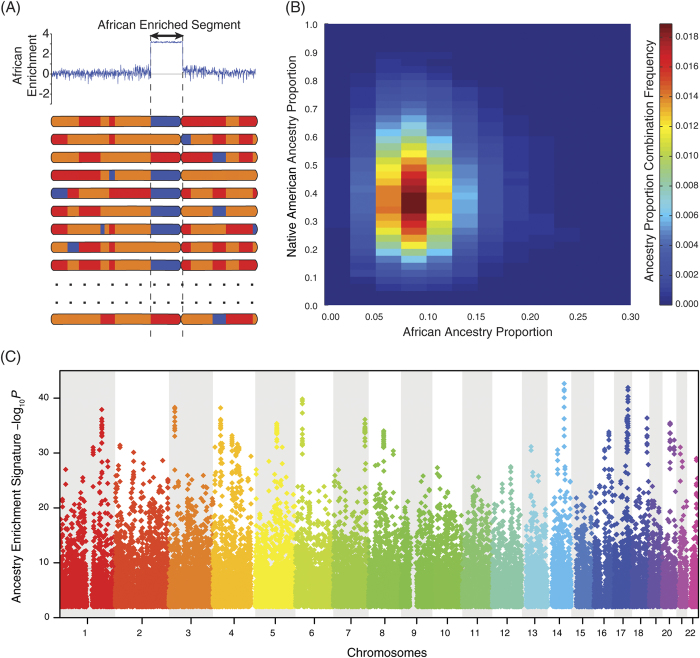
Population-wide ancestry enrichment for Colombian genomes. (**A**) Schematic of the ancestry enrichment technique. Ancestry-enriched regions are identified as genomic loci with anomalously high ancestry contributions, for the entire population, from one of the three continental components. (**B**) Heatmap showing the population frequencies of three-way ancestry proportion combinations for Colombian genomes. Each block corresponds to a specific three-way ancestry proportion combination, and the frequency of that combination in the population is color-coded as shown in the scale adjacent to the heatmap. Note that since there are three continental ancestry components, the value of the third ancestry component (European) is dependent on the first two and thus not shown. (**C**) Manhattan plot showing the genomic regions identified as ancestry-enriched via the trinomial probability (y-axis) of observing a particular three-way ancestry combination in the population (see Materials and Methods).

The rationale of the ancestry enrichment approach is illustrated in Fig. 4A; ancestry-enriched segments are identified by virtue of having anomalously high levels of a specific ancestry contribution compared with the expected values based on population-wide average ancestry proportions. The frequencies of the different three-way ancestry proportion combinations in the Colombian population are represented as a heatmap in Fig. 4B. More common ancestry proportion combinations are shown as hot (red) regions, whereas the cold (blue) regions show less likely (*i.e.* anomalous) ancestry proportion combination values. Chromosomal segments that bear such anomalous ancestry proportion combinations are the ones that are identified by the ancestry-enrichment statistical test applied here (see Materials and Methods). When the ancestry-enrichment analysis technique is applied to population-wide ancestry proportion combination data, numerous statistically significant ancestry-enriched chromosomal segments are revealed across the entire genome (Fig. 4C). A list of all ancestry-enriched chromosomal segments, along with the genes that lie therein, is provided as Supplementary Table S1.

### Health and selection related genes in ancestry-enriched regions

Having identified genomic loci with anomalous ancestry contribution levels, we then interrogated the genes located in these regions for previously identified signatures of natural selection and for associations with health-related traits. Only ancestry-enriched chromosomal segments that were highly statistically significant (*P* < 10^−9^, FDR q-value 6.5 × 10^−9^) were used for these analyses. There are numerous genes in these regions that were previously identified to show evidence of positive selection in populations from one of the three ancestral regions that contributed to the modern admixed Colombian population; there are also a number of genes in ancestry-enriched regions that show evidence for a role in various health-related phenotypes based on previous selection and association studies (Table 2). In particular, there are several immune system related genes that have been previously characterized as subject to positive selection found in ancestry-enriched regions (*CD226*, *HLA-B*, *MICA* and *MAPK10*).

**Figure 5:**
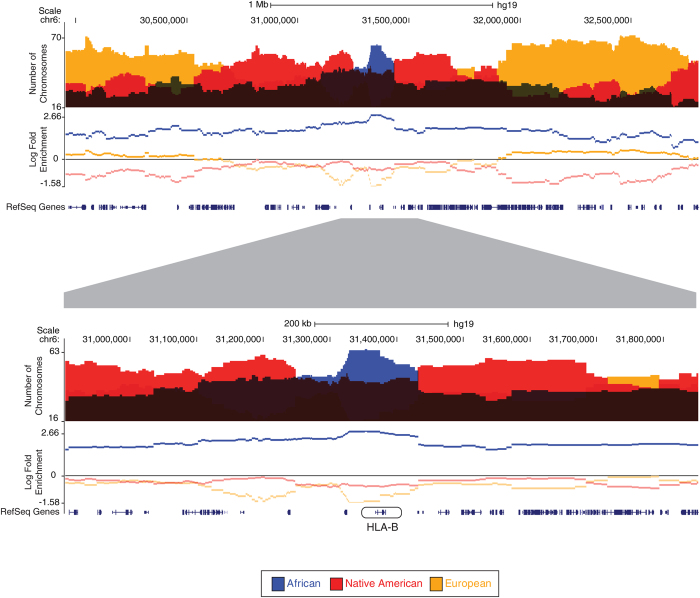
Example of an African-enriched region found in the MHC locus. Population-wide chromosome counts from the three ancestry-components are shown above the genomic axis and log-fold enrichment values (observed counts/genomic average counts) for the ancestry components are shown below the axis. The upper panel shows a ~2.5 Mb region of the MHC locus on chromosome 6, and the lower panel shows a zoomed in view centered on the African ancestry-enriched gene *HLA-B*.

The *HLA-B* gene located in the major histocompatibility complex (MHC) on chromosome 6 is found in an African ancestry-enriched chromosomal segment (Fig. 5). *HLA-B* is an MHC class I gene, which encodes a cell surface protein that presents foreign peptides (antigens) to immune system cells. *HLA-B* is a highly diverse gene with numerous allelic variants; high sequence diversity of *HLA* genes is thought to facilitate the ability to counter a diverse repertoire of foreign antigens. Indeed, *HLA* gene diversity levels among global populations are positively correlated with regional-specific levels of pathogen richness, with African populations having the most diverse repertoire of *HLA* alleles^17^. In addition, *HLA-B* allelic diversity levels world-wide show a strong positive correlation with malaria selective pressure^30^. Enrichment for African alleles of the *HLA-B* locus may have helped the Colombian population to more effectively counter malaria and/or other pathogenic agents. The *MAPK10* gene is another immune-related gene that is located in an Asian-enriched ancestry segment in the Colombian population, and alleles of this gene were recently shown to be selected for malaria resistance in Malaysia^31^.

Some other interesting genes from ancestry-enriched regions include *SCL4A5* and *EDAR*. The *SCL4A5* gene is located in a genomic region enriched for European ancestry in the Colombian population. Positive selection at this locus has previously been associated with the evolution of decreased melanin skin pigmentation as European and Asian populations radiated out of Africa^12^. Light skin has been proposed to have evolved via sexual selection based on male preferences for lighter-skinned mates^32^, as originally proposed by Darwin, although this hypothesis has been contested^33^. It is interesting to speculate as to whether enrichment for European ancestry at this locus can be attributed to preference for lighter-skinned females in Colombia.

The *EDAR* gene has received considerable attention recently owing to an interesting connection between the phenotype it encodes and evidence of population-specific positive selection^9^,^34^. *EDAR* encodes a cell surface receptor that is involved in the development of hair follicles and cutaneous glands, and there is evidence for positive selection of *EDAR* alleles in Asian and Native American populations. The selected allele reduces evaporation from exposed facial structures and upper airways, which is thought to represent an adaptation to cold and dry environments in East Asia. In the admixed Colombian population, *EDAR* is found in a chromosomal segment that is depleted for Asian (Native American) ancestry (Supplementary Fig. S4). This could reflect the fact that the *EDAR* adaptation to cold and dry environments would confer a disadvantage in the hotter and more humid tropical environment of Colombia.

### Immune related pathway genes in ancestry-enriched regions

**Figure 6:**
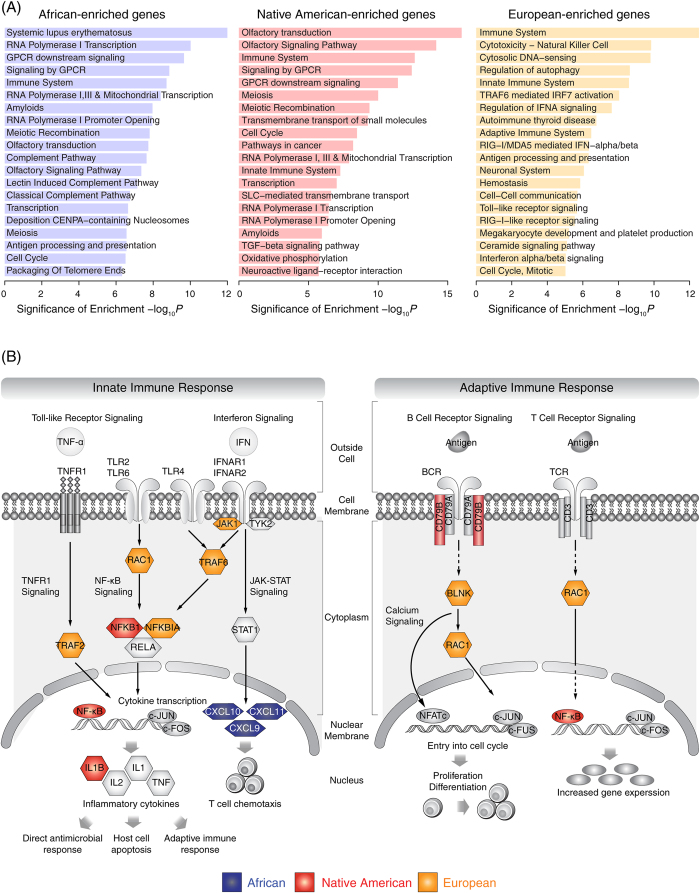
Functional enrichment analysis for ancestry-enriched regions. (**A**) Pathways identified as over-represented among ancestry-enriched regions via gene set enrichment analysis. (**B**) Schematic of four such pathways that are involved in innate and adaptive immune response.

In addition to the targeted analysis approach for the interrogation of genes located in ancestry-enriched regions that yielded the examples described above, functional enrichment analysis was used to evaluate whether specific pathways are over-represented among ancestry-enriched genes. To do this, gene set enrichment (GSEA)^35^ pathway analyses were conducted separately for genomic loci that show African, Asian (Native American) and European enriched ancestry contributions. A number of pathways show up as over-represented when compared against ancestry-enriched genes (Fig. 6A), and similar pathways are found among the different ancestry components despite the fact that their gene sets are mutually exclusive by operational definition. In particular, immune-related pathways consistently appear as significantly over-represented for all three of the ancestry components.

The significant over-representation of ancestry-enriched genes among components of the immune system includes genes that map to pathways involved in both the innate and adaptive immune response (Fig. 6B). The related Toll-like receptor and interferon signaling pathways of the innate immune response contain numerous ancestry-enriched genes, including cytoplasmic members of the NF-κB and JAK-STAT signaling cascades. There are also ancestry-enriched genes that encode downstream members of these pathways, including transcription factors (NF-κB), inflammatory cytokines (IL1B) and chemokines (CXCL9, 10 and 11), which together help direct antimicrobial responses via mechanisms such as host cell apoptosis and T-cell chemotaxis. Ancestry-enriched genes of the adaptive immune response encode members of the B-cell and T-cell receptor signaling pathways, including antigen receptors (CD79B) and cytoplasmic signaling molecules (BLNK and RAC1) along with the NF-κB transcription factor. These proteins help facilitate the proliferation and differentiation of B-cells and T-cells in response to specific immune challenges. Interestingly, these pathways contain a mix of ancestry-enriched genes from different ancestral components. This indicates that individuals from the admixed Colombian population have assembled immune system pathways that are made up of combinations of ancestry-specific alleles that have never been seen in the same genetic background. This may have provided a mechanism to confront the novel combinations of microbial pathogens found in the New World. It should be noted that gene function tends to be spatially correlated, and this is particularly true for the kinds of immune genes discussed here. As this could lead to a violation of the assumptions of independence that underlie GSEA pathway analysis, the exact *P*-values reported here should be taken with some caution.

## Discussion

### Genetic ancestry in Colombia

Results from our analysis of 60 Colombian genome sequences from Medellin point to high levels of three-way continental admixture, with contributions from African, Native American and European ancestral source populations, consistent with previous genetic studies^19^,^20^,^21^,^22^,^23^,^25^ and with overall demographic trends in Colombia (Table 1). On average, admixed genomes from Medellin show predominantly European ancestry; the average genome sequence shows 74.6% European, 18.1% Asian (Native American) and 7.3% African ancestry (Fig. 1D). However, individuals from Medellin vary widely with respect to their ancestry proportions from these three ancestral source populations. There are a number of individuals with >95% European ancestry on one end of the spectrum and people with far more even three-way contributions from the ancestral populations on the other end (Fig. 1C).

It should be noted that different regions of Colombia show very distinct demographic patterns. For example, whereas people from Medellin have primarily European ancestry, the Atlantic and Pacific coastal regions are home to much larger populations of Afro-Colombians. Thus, the admixture patterns reported here for Medellin can not be taken to represent country-wide patterns of Colombian genetic ancestry. Indeed, studies on the genetic ancestry of Colombians sampled from different regions of the country often yield very different results^19^,^20^,^21^,^22^,^23^,^25^. A paper that was published while our own work was in preparation may represent the most comprehensive survey of Colombian genetic ancestry to date^24^. In that study, three-way admixture patterns were inferred for 1,659 individuals based on 30 ancestry informative markers. The results of the study underscore the genetic diversity of the Colombian population. The Colombian samples showed the highest levels of average three-way admixture contributions from ancestral populations (60% European, 29% Native American and 11% African) among the five Latin American countries surveyed as well as the greatest extent of geographical variation in genetic ancestry.

### Ancestry and identity in a Colombian population

Interestingly, the genetic ancestry and admixture results obtained here for the population of Medellin can be considered to be at odds with the demographic data for the city. Medellin is considered to have a population that is almost entirely descended from Europeans. In the 2005 census, 93.4% of the population of Medellin was classified as Euro-descendent, whereas 6.5% of individuals identified as Afro-Colombian and only 0.1% identified as Native American^36^. These demographic data are based on self-identification and reflect the ethnic groups that individuals consider themselves to be members of. Thus, it would appear that the vast majority Colombians from Medellin identify as white despite the presence of a substantial fraction of individuals with appreciable levels of Native American and African ancestry. For example, 46 (~77%) of the Colombian individuals studied here have >2% African ancestry. As 93.4% of the population of Medellin self-identifies as white, it is likely that the majority of these individuals (~43) would self-identify as white. If we assume that these 43 individuals occupy the lower end of the distribution of African ancestry, then 72% of self-identified white Colombians from Medellin have >2% African ancestry. By way of comparison, a recent large-scale analysis of genetic ancestry among different ethnic groups in the United States showed that only 1.4% of self-identified European Americans have at least 2% African ancestry^37^. It should also be noted that the Colombian individuals studied here have substantially higher levels of Native American compared with African ancestry. However, it is not possible to perform the same kind of comparison between ethnic self-identification and genetic ancestry with respect to Native American ancestry given the city-by-city ethnic category data provided in the Colombian census^36^. This is because the census does not distinguish between European descendants who self-identify as white versus individuals with a combination of European and Native American ancestry who identify as mestizo at the level of individual cities.

The distinction between genetic ancestry and ethnic self-identification in Medellin may be related to two important cultural concepts rooted in many Latin American societies: Mestizaje and Blanqueamiento^38^. Mestizaje refers to the intentional mixing of different ethnic groups; it is considered to be a critical part of nation-building and cultural identity, for Colombia in particular and across Latin America^39^,^40^. Blanqueamiento refers to the ideology of racial improvement via the “whitening” of the population. While blanqueamiento may have a biological dimension, with respect to the desire to produce whiter offspring, it more often manifests as a social construct. It is in this social sense that blanqueamiento is reflected in ethnic self-identification. If whiteness is implicitly held up as a social ideal, and a progressive-generational trend that a society should aspire to, people may tend to self-identify as white irrespective of their genetic ancestry^41^. The contrast between the genetic ancestry results obtained here and demographic data point to the possibility that the population of Medellin exemplifies such a trend. On the other hand, since the vast majority of individuals studied here (95%) have genomes with majority European ancestry, these individuals may simply choose to identify most closely as white or European descended despite their genetic admixture.

The relationship between genetic ancestry and ethnic self-identification has recently been studied in depth for five Latin American countries^24^. This work confirmed that there are large variations in genetic ancestry within self-identified ethnic groups across Latin American countries and reveals the large extent to which physical appearance influences ethnic self-identification. Skin pigmentation was shown to have a particularly profound effect on ethnic self-identification, but physical traits and appearance were also shown to be poor indicators of genetic ancestry in the same study. Nevertheless, the relationship between genetic ancestry and self-identification was shown to be quite complex. There are consistent, albeit weak, correlations between genetic ancestry and physical traits for Latin Americans, and the extent to which genetic ancestry is over- or under-estimated for ethnic groups varies according to both the ethnic category and the particular ancestry component (African, European or Native American). Surprisingly, self-perception of European ancestry tends to underestimate the measured European genetic ancestry, whereas self-perception of African ancestry over-estimates the extent of African genetic ancestry. Native American self-perception underestimates the extent of genetic ancestry at lower levels of Native American genetic ancestry comparable to those observed in this study.

### Admixture and the conquest of the Americas

Colombian genome sequences also show strikingly asymmetrical patterns of sex-specific admixture with the male lineages dominated by European ancestry and female lineages comprised of more Native American ancestry (Fig. 2). These results are consistent with a number of previous studies that show similar sex-specific admixture patterns for Colombians^20^,^21^,^22^,^23^,^25^. But we show here for the first time that the pattern of sex-specific ancestry is more pronounced for the Colombian population than for a number of other Latin American countries. This pattern of genetic ancestry reflects the harsh realities of ‘La Conquista’ and the colonial period that followed. Spanish conquistadors were professional warriors who arrived without their families (wives), and subsequent groups of settlers also included relatively small numbers of women^21^. In addition, armed conflicts between conquistadors and indigenous groups resulted in the extermination of a large part of the Native American population, and men were often specifically targeted for elimination^42^. Together, these demographic and historical factors resulted in European males often having children with indigenous women, accounting for the observed sex-specific admixture trend.

### Admixture, fitness and selection in Colombia

When regional-specific ancestry contribution patterns are compared across the entire population of Colombian genomes studied here, it becomes very clear that the patterns of admixture are non-random (Fig. 4). There are numerous ancestry-enriched chromosomal segments that have anomalously high (low) levels of ancestry from one of the three ancestral source populations. These results suggest the possibility that loci enriched for distinct ancestry have been differentially retained in the modern admixed Colombian population owing to their utility in the local environment.

We envision that the particular ‘selection’ process that occurred for the admixed Colombian population, based on assortment among pre-existing ancestry-specific alleles, was somewhat distinct from the concept of natural selection as typically formulated. Adaptive, also referred to as positive or directional, natural selection is most often considered to occur via the fixation of novel mutations based on differential reproductive success. This process starts with the introduction of new alleles by mutation at very low population frequencies and thus is relatively slow; it typically takes place on the order of tens- or hundreds-of-thousands of years^3^. The evolutionary process that yielded ancestry-enriched segments in the admixed Colombian population was instead based on selection among pre-existing population-specific alleles. These population-specific (or enriched) alleles evolved *in situ* in their ancestral regions, based on local selective pressures, over many thousands of years. When the populations that bore these alleles arrived in the New World, the pre-existing adapted alleles were ready to be rapidly re-assorted into novel admixed genomes. In other words, the long, slow process of natural selection had already occurred in the ancestral source populations to generate a standing pool of genetic variation with a wide variety of adaptive utility. From this existing pool of genetic variation, numerous ancestry-specific segments containing adaptive alleles were readily accessible for re-assortment. In this way, the enrichment of ancestry-specific segments in the Colombian population, based on their utility in the New World environment, could have occurred much more rapidly, *i.e.* within the relatively short time span following Columbus’ arrival in the Americas. The ability to rapidly shape admixed genomes over such a time span, with anomalous combinations of ancestry-specific alleles, is supported by the highly asymmetrical patterns of sex-specific admixture observed for Latino populations.

A similar approach to search for evidence of selection based on ancestry-enrichment has been conducted in two recent studies of admixed African American populations^43^,^44^. Results from these two studies differ starkly. The first study reported six highly significant ancestry enriched regions genome-wide and took this as evidence of admixture driven natural selection in African Americans^43^. The more recent study included a substantially larger sample size along with a stringent control for multiple statistical tests and was not able to replicate the previous finding of significantly enriched ancestry regions^44^. One of the potential limitations of the approach that we use here is the far lower sample size compared with previous studies that employed genotyping arrays (SNP chips). It is possible that the ancestry enriched regions reported here will not be replicated when larger sample sizes are used, as was the case for African Americans. However, our approach does have the advantage of increased resolution for local ancestry inference provided by whole genome sequence comparison, which also necessitates relatively low samples sizes compared to genotype studies. We also employ a different statistical approach for the identification of ancestry enriched regions than used in previous studies, which allows us to look for deviations from expected three-way ancestry patterns. This approach is applied using a stringent control for multiple statistical tests. It is also worth noting that the Colombian population analyzed here, and Latino populations in general, show far more admixture than seen for the African American populations analyzed in the aforementioned studies. It is possible that higher levels of admixture provide more opportunities for the differential retention of previously selected ancestral alleles.

### Admixture and the infectious disease burden in Colombia

Interrogation of the functional roles of genes located in the anomalously admixed genomic regions detected here suggests that the retention of ancestry-specific alleles in the Colombian population is most closely tied to immune system function (Table 2 and Fig. 6). This process was likely to have been driven by exposure of the admixed population to numerous microbial pathogens, many of which had never before been encountered. These new pathogens could have come from the tropical environment found in the New World, and/or they could have resulted from the mingling of the three distinct continental population groups, each with their own set of endemic pathogens.

Colombia is located in the tropics, traversed by the equator, and has a high burden of infectious disease caused by a variety of pathogenic agents. Compared with other countries in the region and around the world, Colombia has a very high level of pathogen richness as measured by the number of known pathogens in the country (n = 244)^17^,^45^. The infectious disease burden in Colombia includes waterborne diseases, such as cholera, and vectorborne diseases, including yellow fever, dengue fever and malaria^18^. Host genetic factors play an important role in shaping individuals’ susceptibility and resistance to these pathogenic agents^46^. Natural selection has been shown to increase the frequency of alleles that provide increased resistance to cholera, dengue fever and malaria. In addition, many of these alleles are population-specific (or enriched) having evolved within populations from the geographic regions where the diseases originated.

Susceptibility to cholera, which is endemic to South East Asia, is known to be strongly influenced by host genetic factors^47^,^48^. These include genes that encode members of the NF-κB pathway that operates as part of the innate immune system^49^,^50^. Results from our analysis show this pathway to be enriched for genes with anomalous ancestry patterns including genes that fall within Asian (Native American) enriched segments (Fig. 6).

There have also been numerous studies that have identified host genetic factors that appear to mitigate susceptibility to dengue fever^51^. These include large scale genome-wide association studies (GWAS)^52^,^53^,^54^ along with smaller scale candidate gene studies^55^,^56^,^57^. A recent GWAS study found the most significant dengue disease association SNPs at the *MICB* locus along with additional significant SNPs at the adjacent *HLA-B* and *HLA-C* loci^52^. All of these genes are members of the MHC locus that encodes numerous cell surface receptor proteins that mediate immunity via the presentation of antigen peptide sequences. All three genes are found within African ancestry-enriched segments (Table 2 and Supplementary Table S1). A number of other candidate gene studies have also uncovered associations between specific *HLA* gene alleles and susceptibility to dengue in Asian populations^55^,^56^,^57^.

As of this time, there are no known host resistance alleles associated with susceptibility to yellow fever. However, it has long been thought that populations with different genetic ancestries had differing degrees of susceptibility to yellow fever, suggesting a genetic component to susceptibility to this pathogen. In particular, populations with African ancestry were thought to have reduced susceptibility to yellow fever^58^,^59^,^60^, and this notion has been related to an increased economic incentive for the use of African slave labor in the New World^61^. However, this idea has been criticized based on a lack of historical consensus and the absence of direct evidence for a genetic ancestral component to resistance as is seen for malaria^62^. Nevertheless, a recent analysis of mortality from yellow fever in 19^th^ century United States provides compelling statistical evidence for an association between genetic ancestry and susceptibility to yellow fever^63^. Individuals with African ancestry were found to have indistinguishable incidence rates of yellow fever compared with Europeans but had significantly lower rates of mortality. The authors went on to rule out a number of environmental (*e.g.* social and/or economic) factors that could explain this difference and concluded that their findings support the existence of host genetic factors that mitigate the severity of yellow fever infections among individuals with African ancestry.

Malaria poses a particularly grave and consistent threat to public health in Colombia with both endemic and epidemic transmission regimes^64^. While Medellin does not have a high incidence of malaria owing to its altitude, malaria is endemic to nearby regions at lower altitudes, particularly in jungle areas along the Atlantic and Pacific coasts^65^. As the population of Medellin is cosmopolitan^36^, having consistently received immigrants from surrounding regions over the centuries, malaria may have exerted a selective pressure on the population. Numerous alleles at different genetic loci have evolved under the influence of selective pressure based on resistance to malaria^46^. This process has occurred independently in different populations around the world giving rise to a number of ancestry-specific resistance alleles. For example, there are distinct African- and Asian-specific alleles of the Hemoglobin Beta Chain encoding gene (*HBB*) that evolved independently and provide increased resistance to malaria^66^,^67^,^68^.

There is a clear connection between the infectious disease burden in Colombia, for a number of the most common pathogen agents found in the country as described above, and host genetic factors that mediate susceptibility and resistance. Many of these host genetic factors are likely to be population-specific (or enriched) alleles that initially evolved within ancestral source populations. The results obtained from our ancestry-enrichment analysis indicate that the modern admixed Colombian population may have had the opportunity to draw from a variety of these ancestry-specific resistance alleles to assemble a diverse repertoire of immune system related genes capable of countering threats from the wide variety of pathogens found in the New World environment. In addition to being of interest from an evolutionary perspective, these results may have implications for better understanding the genetic determinants of health in highly admixed Latin American populations.

## Materials and Methods

### Genome sequence data

A total of 581 whole genome sequences or genotypes, taken from a variety of sources, were analyzed here (see Supplementary Fig. S1). Whole genome sequences for 60 admixed Colombians from Medellin were taken from the 1000 Genomes Project^26^,^27^. Whole genome sequences (*n* = 264) and genotypes characterized using SNP microarrays (*n* = 257) from a number of sources^25^,^26^,^27^,^69^ were analyzed for putative ancestral populations in Africa, the Americas, Asia and Europe. Genotypes (*i.e.* SNP calls) characterized using complete genome sequences and microarrays from these different studies (and formats) were merged using the program PLINK v1.90^70^, along with custom scripts, in order to create a single set of merged SNPs across all studies. PLINK was then used to perform linkage disequilibrium pruning on the merged SNP data to produce a reduced set of unlinked SNPs [options: —indep-pairwise 100 25 0.05 —mind 1 —geno 0.01 —hwe .001]. These processes were done separately for genome sequence and genotype data together and for genome sequence data alone.

### Ancestry and admixture analysis

Allele sharing distances between pairs of genomes were computed as the fraction of differences between SNP calls. Principal components analysis of the resulting pairwise allele sharing distance matrix was performed using the prcomp program from the R package v3.1.2^71^ [options: scale=TRUE] to relate admixed and ancestral genomes. The program ADMIXTURE v1.23^72^ was used to estimate the admixture fractions of three putative ancestral populations – African, Asian (Native American) and European – among Colombian genome sequences. ADMIXTURE was run with default settings and k = 3 ancestral populations. The program SupportMix (Ver Jul 18 2012)^29^ was used to characterize the regional (locus-specific) three-way ancestry admixture fractions in the Colombian genomes using default settings. Locus-specific ancestry admixture analysis was done using whole genome sequences from the 1000 Genomes Project (Supplementary Fig. S1) to afford increased resolution.

### Sex-specific admixture contributions

Normalized ratios of the difference between admixture fractions for the X chromosome versus the autosomes were used to infer sex-specific admixture contributions for each of the three ancestral source populations. The admixture difference ratio (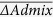) for each ancestry was calculated as:


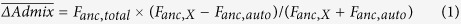


where *F*_*anc,total*_ is the genome-wide admixture proportion for a given ancestry, *F*_*anc,X*_ is the X chromosome admixture proportion for a given ancestry and *F*_*anc,auto*_ is the autosomal admixture proportion for a given ancestry. Positive values of 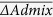 are indicative of an excess of female-specific (maternal) admixture contributions, whereas negative values indicate an excess of male-specific (paternal) admixture. The normalized X chromosome versus autosome ratio values were computed for Colombian genomes along with previously reported genome data from four other Latin American countries^25^. A bootstrapping procedure (1000 replicates) was used to create pseudo-replicate data sets to compute 95% confidence intervals for the observed 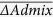 values.

### Ancestry enrichment analysis

Chromosomal regions with anomalous patterns of ancestry, when considered as an ensemble across the entire population of 60 Colombian genomes (120 chromosomes), were identified via ancestry-enrichment probability values. These values were computed as the trinomial probability of observing a particular numerical combination of African (*YRI*), Asian (*CHB*) and European (*CEU*) chromosomal segments, for the entire population at a specific genomic locus, given the population-average levels of African, Asian and European ancestry. This probability is expressed as:


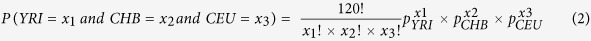


where *x*_1_, *x*_2_, *x*_3_ are the observed chromosomal segment counts at a particular locus with *YRI*, *CHB* and *CEU* ancestries, and 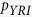, 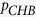, 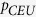 are the expected probabilities based on the population-average ancestry values.

Genomic regions with highly statistically significant (*P* < 10^−9^, FDR q-value 6.5 × 10^−9^) anomalous patterns of ancestry identified in this way were interrogated for their potential functional significance using several sources of information. Genes that lie within these regions were compared to sets of genes previously identified as being subject to positive (adaptive) selection in a number of studies^6^,^7^,^8^,^9^,^10^,^46^, and to genes implicated in human health/disease via association studies using literature surveys and the NHGRI GWAS catalog (accessed May 2014)^73^. The functional affinities of genes encoded in these regions were also evaluated with gene set enrichment analysis using the GSEA software web v3.87^35^.

## Additional Information

**How to cite this article**: Rishishwar, L. *et al.* Ancestry, admixture and fitness in Colombian genomes. *Sci. Rep.* **5**, 12376; doi: 10.1038/srep12376 (2015).

## Supplementary Material

Supplementary Figures 1-4

Supplementary Video

Supplementary Dataset 1

## Figures and Tables

**Table 1:** Demographic profile of ethnic groups for Colombia and Medellin.

**Ethnic Group**	**Colombia (specific)**^1^	**Colombia (broad)**^1^	**Medellin (broad)**^2^
Mestizo (Amerindian and European)	58%	78%	93.4%
White (European)	20%		
Mulatto (Black/African and European)	14%	21%	6.5%
Black (African)	4%		
Zambo (Amerindian and Black/African)	3%		
Amerindian	1%	1%	0.1%

^1^Colombian ethnic group percentages are taken from the CIA World Factbook^18^. Specific and broad ethnic groupings are shown.

^2^Medellin ethnic group percentages are taken from the Colombian census^36^. Only broad ethnic groupings are available for individual cities.

**Table 2:** Genes in ancestry-enriched regions and their associated traits.

**Gene**	**Function**	**Ancestry**	**Associated trait**	**Evidence**	**PMID**	**Ancestry enrichment (-log_10_*P*)**
ADCY3	Adenylate cyclase 3	Asian	Body mass index	Positive Selection	22344219	18.6
ATM	Ataxia telangiectasia mutated	Asian	Cell cycle	Association Study	24390342, 21983787	15.7
BCHE	Butyrylcholinesterase	African	Cardiovascular disease risk in European	Association Study	23419831, 21943158, 9780523	23.7
CASP8	Caspase 8, apoptosis-related cysteine peptidase	Asian	Reduces breast cancer risk	Positive Selection	17293864	9.6
CD226	CD226 molecule	Asian	Adaptive immune system, cell adhesion	Positive Selection	24390342, 23128233, 21829393, 17554260	34.5
HLA-B	Major histocompatibility complex, (MHC) class I, B	African	Immune response	Positive Selection	23731540, 16998491	31.6
MANBA	Mannosidase	Asian	Decreases colorectal cancer risk & associated with mannosidosis	Association Study	17899454, 21833088	29.7
MAPK10	Mitogen-activated protein kinase 10	Asian	Immune system	Association Study	25634076	25.4
MICA	MHC class I polypeptide-related sequence A	African	Immune response	Positive Selection	23731540	32.6
NANOS3	Nanos homolog 3	Asian	Germ cell development	Association Study	21421998	23.9
NFKB1	Nuclear factor of kappa light polypeptide gene enhancer in B-cells 1	Asian	Reduces breast cancer risk	Positive Selection	22562547	30.8
SF3B4	Splicing Factor 3b, Subunit 4	Asian	Height	Association Study	20881960, 18391951	30.6
SLC24A5	Solute carrier family 24, member 5	European	Decreases melanin pigmentation in skin	Positive Selection	17182896	16.1
SLC44A2	Solute carrier family 44, member 2	European	Metabolism	Association Study	22040064	20.1
ULBP1	UL16 binding protein 1	European	Cytosolic DNA sensing pathway	Association Study	20923822	12.3
USP32	Ubiquitin Specific Protease 32	Asian	Overexpressed in breast cancer	Positive Selection	19307593	36.4
