# Supplementary material for: Ancestry, admixture and fitness in Colombian genomes
Source: Sci Rep. 2015 Jul 21;5:12376. doi: 10.1038/srep12376 (PMC4508918; doi:10.1038/srep12376)
Supplement: Supplementary Figures 1-4 [file srep12376-s1.pdf]

Supplementary Information for:

Ancestry, admixture and selection in Colombian genomes

Lavanya Rishishwar[1,2,3,†], Andrew B. Conley[1,†], Charles H. Wigington[1], Lu Wang[1], Augusto Valderrama-Aguirre[2,4,5] and I. King Jordan[1,2,3,*]

[1]School of Biology, Georgia Institute of Technology, Atlanta, GA 30332, USA
[2]PanAmerican Bioinformatics Institute, Cali, Valle del Cauca, Colombia
[3]BIOS Center de Bioinformática y Biología Computacional, Manizales, Caldas, Colombia
[4]Biomedical Research Institute, Universidad Libre, Cali, Valle del Cauca, Colombia
[5]Regenerar - Center of Excellence for Regenerative and Personalized Medicine, Valle del Cauca, Colombia

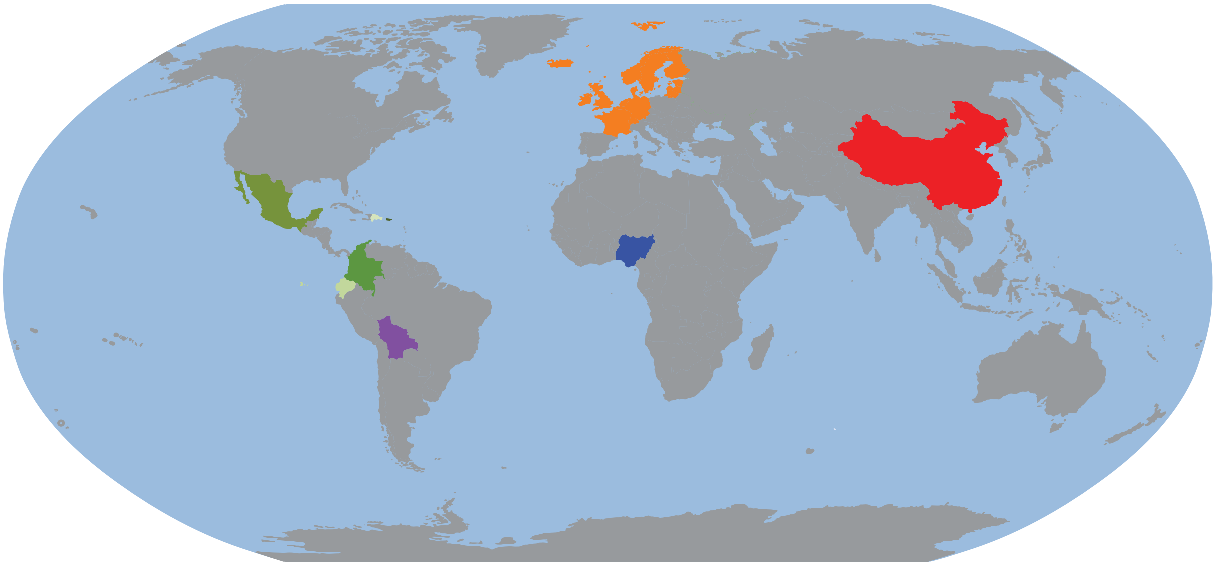

Ancestral Populations from Around the World

Name	Color	Ancestral Origin	n	Source (PMID)	Platform
CEU		Northern & Western Europe	85	23128226	Whole genome sequencing
CHB		Beijing, China	92	23128226	Whole genome sequencing
Totonac & Bolivian		Mexico & Bolivia	45	22606979	SNP microarray
YRI		Nigeria	87	23128226	Whole genome sequencing

Admixed Populations from the Americas

Name	Color	Country	n	Source (PMID)	Platform
CLM		Medellin, Colombia	60	23128226	Whole genome sequencing
Colombian		Colombia	26	20445096	Illumina 610K array
Dominican Republican		Dominican Republic	27	20445096	Illumina 610K array
Ecuador		Ecuador	20	20445096	Illumina 610K array
Mexican		Mexico	112	20445096	Affymetrix GeneChip 500K Array Set
Puerto Rican		Puerto Rico	27	20445096	Illumina 610K array

S1 Figure. World-wide source of genome sequence and genotype data analyzed in this study. The global locations of putative ancestral populations are shown – African (blue), Asian (red), European (orange) and Native American (purple) – along with the locations of admixed American populations (green). Information on the source populations, number of samples, supporting publication and genome characterization platform is provided.

Note: The global map shown here was modified from http://commons.wikimedia.org/wiki/File:World_Map_Blank_-_with_blue_sea.svg, which has been released into the public domain and thus free to be used by anyone for any purpose.

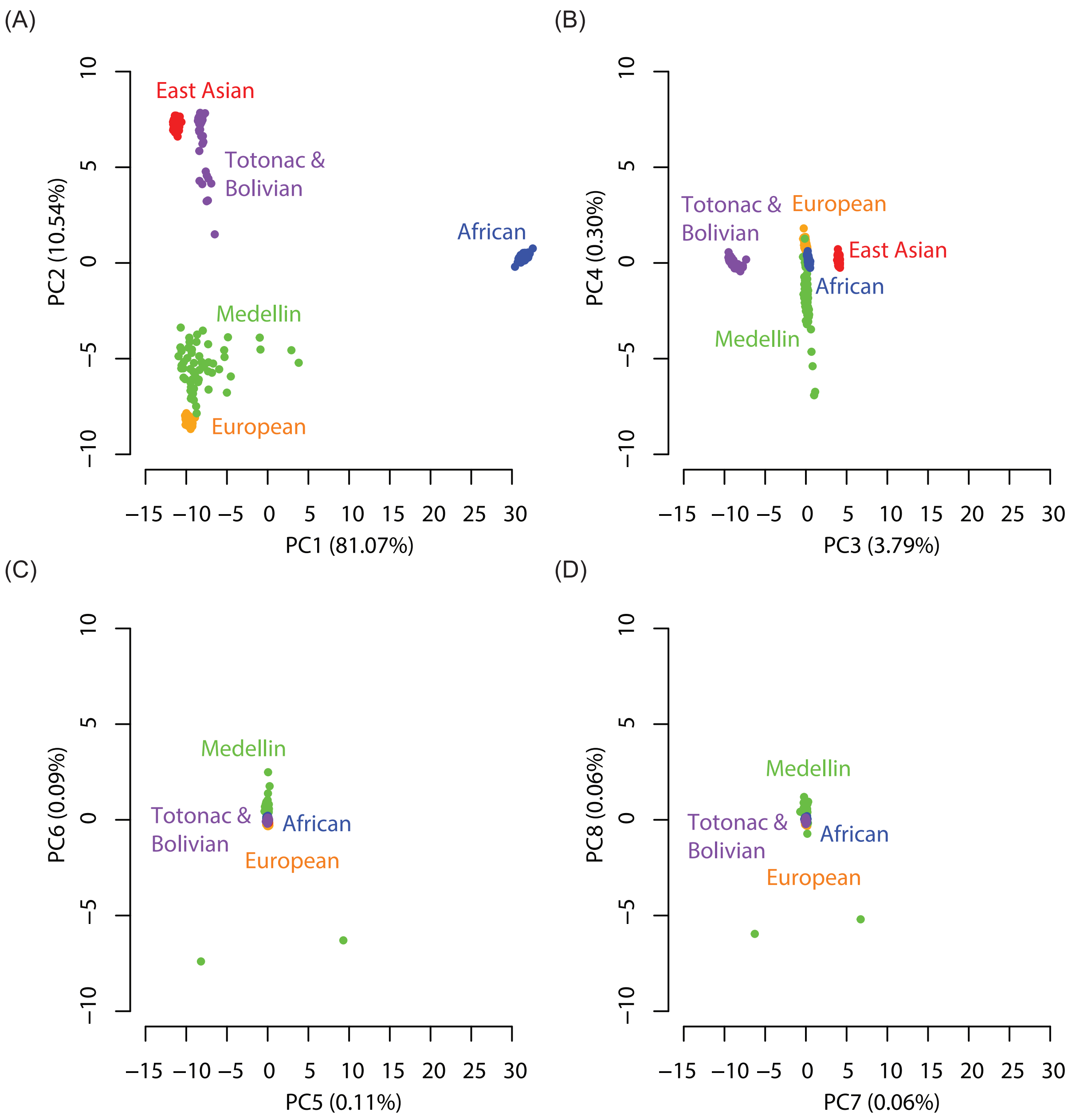

S2 Figure. PCA of pairwise allele sharing distances among admixed Colombian genomes from Medellin compared to putative ancestral populations from Africa, the Americas, Asia and Europe. The panels have comparisons of the successive principal components (PCs): (A) PC1-PC2, (B) PC3-PC4, (C) PC5-PC6 and (D) PC7-PC8.

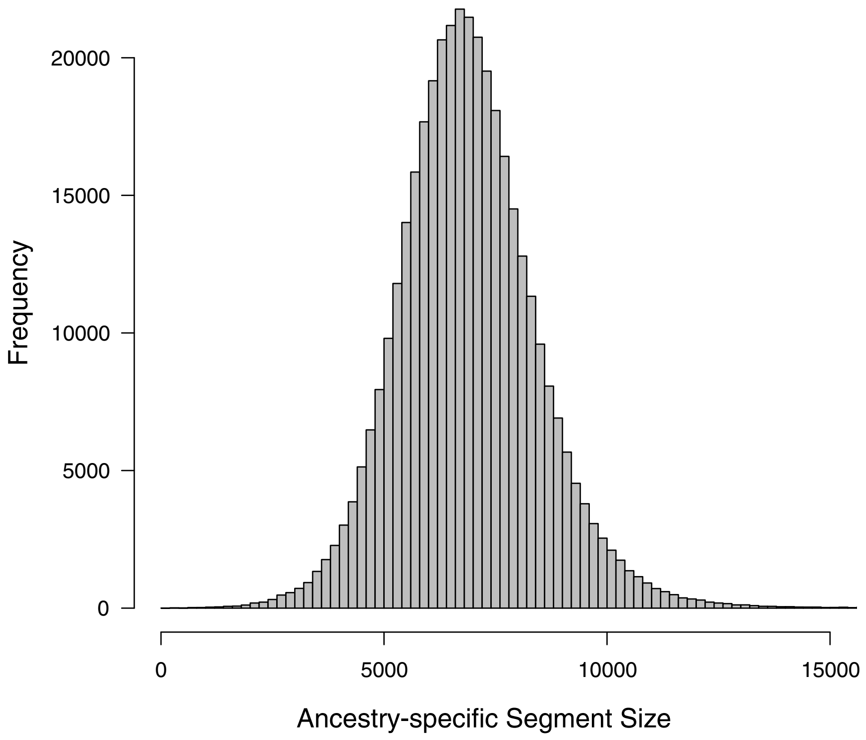

S3 Figure. Size distribution of the ancestry-specific chromosomal segments used for the ancestry enrichment analysis.

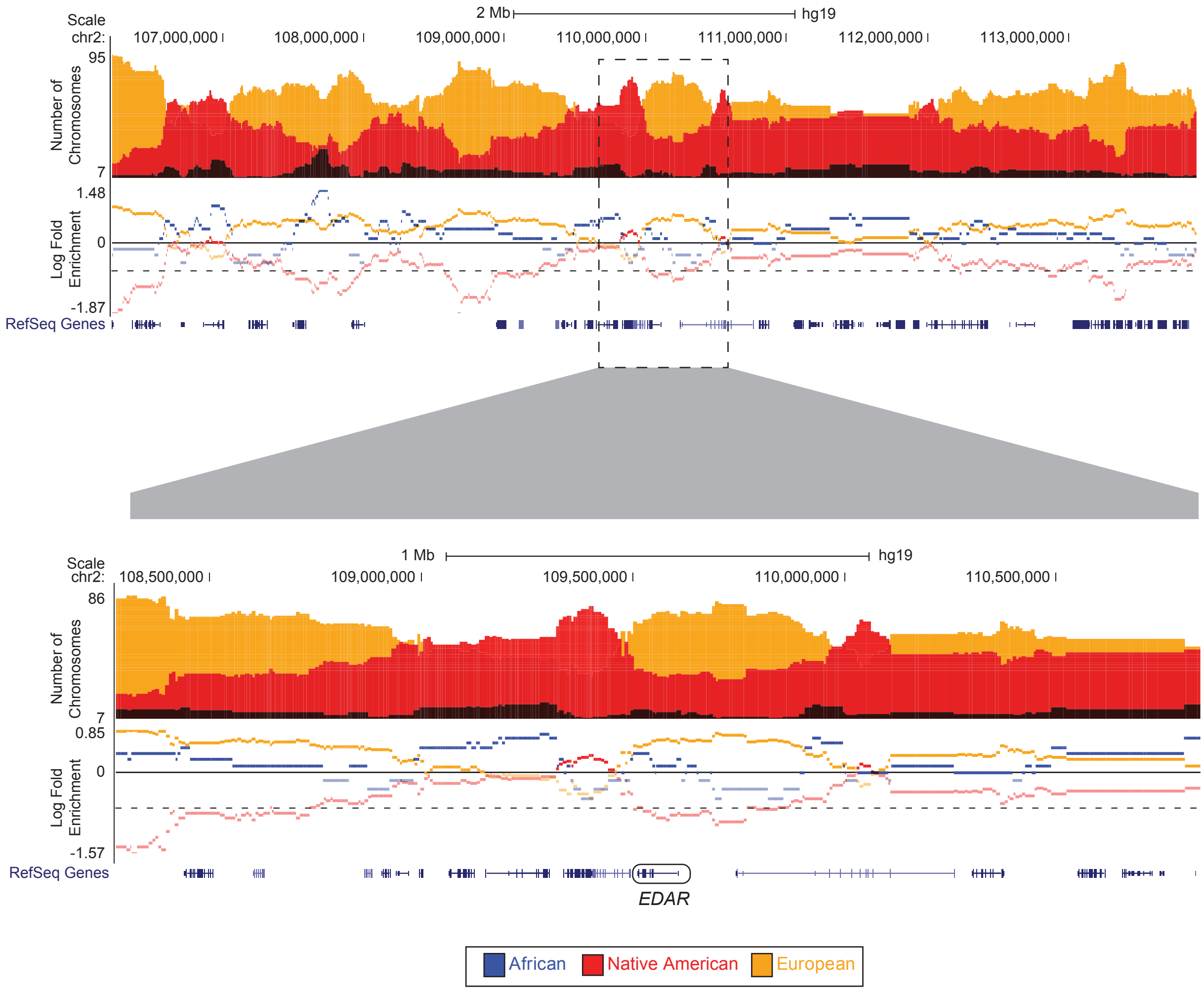

S4 Figure. Example of an Asian (American)-depleted region found at the EDAR locus. Population-wide chromosome counts from the three ancestry-components are shown above the genomic axis and log-fold enrichment values (observed counts/genomic average counts) for the ancestry components are shown below the axis. The upper panel shows a ~7Mb region surrounding the locus on chromosome 2, and the lower panel shows a zoomed in view centered on the Asian (American) ancestry-depleted gene EDAR.
